# Impact of mutations on pregnancy outcome in patients with myeloproliferative neoplasms

**DOI:** 10.1002/jha2.622

**Published:** 2022-12-07

**Authors:** Dawn Maze, Iyad Arusi, Vikas Gupta, Eshetu G. Atenafu, Ann Kinga Malinowski, Nadine Shehata

**Affiliations:** ^1^ Elizabeth and Tony Comper MPN Program Princess Margaret Cancer Centre University of Toronto Toronto Ontario Canada; ^2^ Division of Hematology Mount Sinai Hospital Special Pregnancy Program University of Toronto Toronto Ontario Canada; ^3^ Princess Margaret Cancer Centre University of Toronto Toronto Ontario Canada; ^4^ Department of Obstetrics and Gynaecology Division of Maternal‐Fetal Medicine Department of Obstetrics and Gynaecology, Lunenfeld‐Tanenbaum Research Institute Mount Sinai Hospital University of Toronto Toronto Ontario Canada

**Keywords:** MPN, pregnancy, somatic mutation

## Abstract

The impact of driver and other somatic mutations on pregnancy outcomes is unknown. The purpose of this study was to report the management and outcome of pregnancies in a cohort of myeloproliferative neoplasms (MPN) patients, particularly to evaluate the impact of somatic mutations. The cohort included consecutive patients with MPN who had a least one confirmed pregnancy. The primary outcome was live births. Secondary outcomes were thrombotic and major bleeding events. Between 2010 and 2021, 29 pregnancies occurred in 24 individuals with MPN. Aspirin was used in 24 cases (83%) and interferon alfa in five (17%). There were 24 live births (83%). There were three thrombotic events, two antepartum and one postpartum. Miscarriages and thrombotic events occurred in JAK2‐mutated and triple negative, but not CALR‐mutated, MPN. Additional somatic mutations were rare, and there were no apparent associations with pregnancy loss or complications. While JAK2 V617F is associated with an increased risk of thrombosis, its impact on pregnancy outcome has been inconsistently reported. The association between triple negative MPN and adverse pregnancy outcome has not previously been reported. While limited by small numbers, this study underscores the importance of describing driver and other mutations to direct optimal antenatal care in individuals with MPN.

## 1 INTRODUCTION

Myeloproliferative neoplasms (MPN) result from constitutive activation of the janus kinase/signal transducer and activator of transcription (JAK/STAT) signaling pathway and are characterized by proliferation of differentiated myeloid cells, constitutional and other disease‐related symptoms, increased risk for thrombotic and hemorrhagic events, and a propensity to transform to acute myeloid leukemia. Driver and other somatic mutations influence MPN phenotype, thrombotic risk and risk of disease progression [1, 2, 3, 4]. The effect of these mutations on pregnancy outcomes has not been well described. Pregnancy in patients with MPN is associated with maternal and fetal risk, including venous or arterial thrombotic events, bleeding and placental insufficiency leading to preeclampsia, fetal growth restriction, placental abruption or pregnancy loss. We describe the management and outcome of pregnancies in a Canadian cohort of MPN patients, particularly to evaluate the incidence and impact of somatic mutations.

## 2 MATERIALS AND METHODS

The cohort included consecutive patients with Philadelphia chromosome negative MPN, diagnosed according to the 2016 WHO critieria [5] who had at least one confirmed pregnancy after MPN diagnosis. Pregnancy losses were defined as follows: before 12 weeks’ gestation was considered first trimester loss, between 12 and 28 weeks was second trimester loss, and 28 weeks or later was defined as stillbirth. Patients attended Mount Sinai Hospital and/or the Elizabeth and Tony Comper MPN Program at the Princess Margaret Cancer Centre in Toronto, Canada, university‐affiliated centers. The study was approved by both institutional review boards and performed in accordance with the Declaration of Helsinki.

Demographic and clinical data were obtained from medical records. Mutation analysis was performed on DNA from peripheral blood or bone marrow cells. JAK2 (p.V617F and exon 12) and CALR (exon 9) mutations were identified by florescent multiplex polymerase chain reaction and  amplification‐refractory mutation system followed by fragment analysis on the ABI Genetic Analyzer (Applied Biosystems, Waltham, MA). Other genes were profiled using targeted next‐generation sequencing panels: the amplicon‐based 54‐gene panel (TruSight Myeloid Sequencing Panel, Illumina, San Diego, CA) [6] and a custom hybridization‐based 49‐gene panel (Oxford Genes Technologies, OX), as previously described [7]. Variant analysis was performed as previously described [7].

The primary outcome was live births. Key secondary outcomes were antepartum and postpartum thrombotic events and major hemorrhage. For univariate and multivariate analyses, end points were fit using the Cox proportional hazards model. Regression analyses were performed using SAS version 9.4.

## 3 RESULTS

Between 2010 and 2021, 29 pregnancies occurred among 24 individuals with MPN. Characteristics are described in terms of pregnancies (Table 1). Of the 29 pregnancies, 14 (48%) were in primiparous, and 15 (52%) in multiparous individuals; median number of prior pregnancies 1 (range 0–3). In 12 cases, there was a history of one or more pregnancy losses prior to the diagnosis of MPN. During the index pregnancy, most patients received antenatal low dose aspirin (24/29, 83%). Low molecular weight heparin (LMWH) was used in addition to aspirin in five (17%) pregnancies. Interferon alpha (IFN) was used in five (17%) pregnancies and was the only cytoreductive therapy used antenatally.

**TABLE 1:** Characteristics of pregnancies in women with MPN

	Driver mutation, *n* (%)				
	Full cohort	JAK2	CALR	JAK2, CALR	Triple negative
Pregnancies	29	19 (66)	4 (14)	1 (13)	5 (17)
Median age, years (range)	33 (21–45)	33 (23–45)	35 (28–37)	33	27 (21–37)
MPN diagnosis, *n* (%)					
ET	14 (48)	7 (37)	3 (75)	0 (0)	4 (80)
PV	9 (31)	8 (42)	0 (0)	0 (0)	1 (2)
MF	4 14)	4 (21)	0 (0)	0 (0)	0 (0)
MPN NOS	2 (7)	0 (0)	1 (25)	1 (100)	0 (0)
Additional somatic mutations, *n* (%)					
ASXL	2 (7)	2 (7)	0 (0)	0 (0)	0 (0)
TET2	1 (3)	1 (3)	0 (0)	0 (0)	0 (0)
None	9 (31)	4 (14)	4 (100)	0 (0)	1 (20)
Unavailable	17 (59)	12 (41)	0 (0)	1 (100)	4 (80)
Median BMI, kg/m^2^ (range)	25 (17–35)	27 (16–35)	25 (25–25)	24	22 ( 18–26)
Medical history, *n* (%)					
Smoking	5 (17)	3 (16)	1 (25)	0 (0)	1 (20)
Diabetes mellitus	1 (3)	1 (3)	0 (0)	0 (0)	0 (0)
Dyslipidemia	0 (0)	0 (0)	0 (0)	0 (0)	0 (0)
Prior vascular events, *n* (%)					
Venous thrombosis	4 (14)	3 (16)	0 (0)	0 (0)	1 (20)
Hemorrhage	2 (7)	0 (0)	1 (25)	0 (0)	1 (20)
Portal hypertension	2 (7)	1 (5)	0 (0)	0 (0)	1 (20)
Past obstetric history					
Prior pregnancy, yes (%)	15 (52)	11 (58)	2 (50)	0 (0)	2 (40)
Loss <12 weeks, *n* (%)	5 (17)	4 (21)	1 (25)	0 (0)	0 (0)
Loss 12–28 weeks, *n* (%)	6 (21)	4 (21)	1 (25)	0 (0)	1 (20)
Loss >28 weeks, *n* (%)	2 (7)	2 (11)	0 (0)	0 (0)	0 (0)
Hematological parameters prior to index pregnancy, median (range)					
Leucocytes (x 10^9^/L)	10.3 (5.2–19.4)	10.9 (7.9–9.4)	7.1 (5.2–12.0)	‐	5.31
Hemoglobin (g/L)	131 (100–218)	132 (101–218)	112 (100–119)	‐	139
Hematocrit (%)	0.40 (0.23–0.66)	0.4 (0.4–0.7)	0.34 (0.32–0.37)	‐	0.42
Platelet count (x 10^9^/L)	867 (319–2430)	800 (328–1482)	2242 (319–2430)	‐	950
MPN treatment prior to index pregnancy, *n* (%)					
Aspirin	18 (62)	12 (63)	4 (100)	0 (0)	2 (40)
Warfarin	2 (7)	2 (11)	0 (0)	0 (0)	0 (0)
Hydroxyurea	5 (17)	1 (5)	2 (50)	0 (0)	2 (40)
Interferon alpha	1 (3)	0 (0)	1 (25)	0 (0)	0 (0)
Anagrelide	3 (10)	0 (0)	1 (25)	0 (0)	2 (40)
MPN treatment during index pregnancy, *n* (%)					
Aspirin	24 (83)	19 (100)	3 (75)	0 (0)	2 (40)
LMWH	5 (17)	4 (21)	1 (25)	0 (0)	0 (0)
Interferon alpha	5 (17	2 (11)	3 (75)	0 (0)	0 (0)
Pregnancy outcome					
Live birth, *n* (%)	24 (83)	16 (84)	4 (100)	1 (100)	3 (60)
Gestational age at delivery, weeks (range)	39 (31–40)	39 (33–40)	39 (38–39)	31	39 (38–39)
Weight at delivery (g)	3070 (2050–4010)	3070 (2050–4010)	3208 (3070–3480)	2560	2910 (2760–3450)
Delivery, *n* (%)					
Vaginal delivery	14 (48)	9 (56)	2 (50)	1 (100)	2 (67)
Cesarean section	9 (31)	6 (38)	2 (50)	0 (0)	1 (33)
Unavailable	1 (4)	1 (6)	0 (0)	0 (0)	0 (0)
Antepartum complications, *n* (%)					
Preeclampsia	0 (0)	0 (0)	0 (0)	0 (0)	0 (0)
Thrombotic event	2 (7)	1 (5)	0 (0)	0 (0)	1 (20)
Hemorrhage	0 (0)	0 (0)	0 (0)	0 (0)	0 (0)
Postpartum complications					
Thrombosis	1 (3)	1 (5)	0 (0)	0 (0)	0 (0)
Hemorrhage	1 (3)	0 (0)	1 (25)	0 (0)	0 (0)

Abbreviations: ET, essential thrombocythemia; MF, myelofibrosis; MPN NOS, myeloproliferative neoplasm not otherwise specified; PV, polycythemia vera.

Of the 29 pregnancies, there were 24 (83%) live births. There were three first trimester losses, 1 s trimester loss, and one stillbirth. There was no association between driver mutation and live birth (*p* = 0.3). Three of the losses occurred in pregnancies in which JAK2 was the driver mutation (two first trimester losses and the stillbirth), and two losses occurred in pregnancies with triple‐negative MPN (one first trimester loss and the second trimester loss). No pregnancy losses occurred in cases with a CALR mutation. The stillbirth occurred in a patient who was diagnosed with JAK2‐mutated ET late in the second trimester of pregnancy and started aspirin at 24 weeks. Maternal vascular malformation resulted in severe IUGR and fetal demise at 28 weeks. Of the three losses that occurred in JAK2‐mutated MPN, variant allele frequency (VAF) was available in one case and was 17%. Of the other pregnancies in which JAK2 V617F VAF was available, the levels were 5% and 31%, respectively, and both resulted in full‐term deliveries. Next generation sequencing (NGS) was available in 10 pregnancies and revealed additional somatic mutations in three: an ASXL1 mutation (G1397S) in a case with JAK2‐mutated polycythemia vera (PV) and a different ASXL1 variant (N986S) in a case with JAK2‐mutated ET, both of whom had full‐term deliveries. Two TET2 variants (S1039L, T1093I) were detected in a pregnancy with JAK2‐mutated prefibrotic myelofibrosis (MF) that ended in first trimester loss.

There was no association between medical history and baseline hematologic parameters and fetal loss (Table 2). Of the five pregnancies that ended in loss, four were first pregnancies, and one was a second pregnancy with prior second trimester loss. Low‐dose aspirin was used in four of the cases. IFN and LMWH were not used in any of these pregnancies. Antenatal IFN was used in five pregnancies: two with CALR mutation and extreme thrombocytosis (white blood cell (WBC) above 2000 × 10^9^/L), one with JAK2 mutation and prior thrombosis, and two with JAK2 mutation and prior second trimester pregnancy losses. All of these pregnancies resulted in live, full‐term births.

**TABLE 2:** Association between live birth and baseline medical and hematologic parameters

	Live Birth		
	Number (*n* = 5)	Yes (*n* = 24)	*p*‐Value
Median age, years (range)	32 (21–40)	33 (29–37)	0.40
BMI, median (range)	26.3 (25–35)	24.9 (17–25)	0.29
Hematological parameters prior to index pregnancy, median (range)			
Leucocytes (×10^9^/L)	9.9 (10–10)	10.6 (5–19)	0.82
Hemoglobin (g/L)	115 (115–115)	131.5 (100–218)	0.35
Hematocrit (%)	0.4 (0–0)	0.4 (0–1)	0.38
Platelet count (×10^9^/L)	892 (775–1000)	841 (319–2430)	0.90
Prior thrombosis, *n* (%)	1 (20)	3 (12.5)	0.66
Prior bleeding, *n* (%)	1 (20)	1 (4.6)	0.2

Two pregnancies were complicated by antepartum, and one pregnancy by postpartum, thrombosis. There was one postpartum hemorrhage and no cases of preeclampsia. One of the antepartum thrombotic events occurred in a pregnancy with triple‐negative ET; portal vein thrombosis was diagnosed at 8 weeks’ gestation, and the pregnancy was electively terminated. Prior to this event, the individual had been receiving aspirin 81 mg daily. The other antepartum thrombotic event occurred in a pregnancy with JAK2‐mutated ET. LMWH was started and the pregnancy ended in full‐term live birth. Twenty‐one (72%) patients received LMWH for at least 6 weeks postpartum. One pregnancy with CALR‐mutated ET was complicated by a primary postpartum hemorrhage requiring red cell transfusion. The individual had a baseline, pre‐pregnancy platelet count of more than 2000 × 10^9^/L and had received antepartum IFN, aspirin 81 mg and prophylactic LMWH. The platelet count was more than 800 × 10^9^/L at the time of delivery. There was a postpartum pulmonary embolism in a pregnancy with JAK2‐mutated PV, not treated with postpartum LMWH prophylaxis.

## 4 DISCUSSION

In our cohort of pregnancies in individuals with MPN, 83% resulted in live births. This compares favorably to other series reported in the literature [8, 9], but is lower than that reported in a recent UK prospective cohort series [10]. Additionally, there was 1 stillbirth (3%), which is higher than expected. In a recent population‐based study of pregnancies in patients with MPN, stillbirth was reported in 0.6% [11]. Losses occurred in JAK2‐mutated and triple‐negative MPN. No pregnancy losses occurred in CALR mutated MPN. Additionally, thrombotic events occurred only in pregnancies with JAK2 and triple negative MPN. The one bleeding event, a postpartum hemorrhage, occurred in a patient with CALR‐mutated ET and thrombocytosis at delivery. Additional somatic mutations were rare, and there was no apparent association with pregnancy loss or complications.

The impact of driver mutation on pregnancy outcome has been inconsistently reported with some studies finding an association between a JAK2 mutation and pregnancy complications or fetal loss [12, 13, 14], and others reporting no association [15, 16, 17]. A recent study of 155 pregnancies in patients with ET found no association between mutational status and overall fetal loss but did report a relationship between the JAK2 V617F mutation and late pregnancy loss [16]. In our recent meta‐analysis, there was also no significant difference in pregnancy loss between pregnancies with and without JAK2 V617F (5 studies, *n* = 316) [9], but importantly, studies that combined fetal losses with other pregnancy complications were excluded resulting in exclusion of some studies that have reported an adverse effect of a JAK2 mutation on pregnancy outcomes [12]. Several studies have demonstrated a correlation between JAK2 allele burden and hematologic parameters [17], thrombotic events [18, 19], and disease progression [20]. In this study, there was no apparent association between allele burden and pregnancy losses or thrombotic events. While triple negative MF is associated with poorer leukemia‐free and overall survival [3], the same association has not been demonstrated in ET [21]. An association between triple negative MPN and adverse pregnancy outcome has not previously been reported and merits further study.

Limitations of this study include the small sample size, missing data due to retrospective design, and the possibility that early losses may not have been recognized and thus be underestimated. This study nonetheless emphasizes the need for describing driver and other somatic mutations and their associations with maternal and fetal outcomes to direct optimal antenatal management.

## AUTHOR CONTRIBUTIONS

DM and NS designed the project. IA collected the data. All authors contributed to preparing the manuscript.

## CONFLICT OF INTEREST

The authors have no relevant conflict to declare.

## ETHICS STATEMENT

This study was research ethics board‐approved and performed in accordance with the Declaration of Helsinki.

## Data Availability

The data that support the findings of this study are available from the corresponding author upon reasonable request.
